# A Proposition

**Published:** 1884-04

**Authors:** Frank Abbott, J. E. Cravens, C. T. Stockwell, F. Searle, W. C. Wardlaw, B. H. Catching

**Affiliations:** New York City; Indianapolis, Ind.; Springfield, Mass.; Springfield, Mass.; Augusta, Ga.; Atlanta, Ga.


### A Proposition.

*To the Presidents and Members of State Dental Associations:*

Gentlemen--The foremost men of our profession admit the fact that the name of American Dentistry has not that world-wide recognition which its honor demands, and its history would warrant. In our opinion, this is an opportune time for a general movement to place it upon a basis commensurate with its dignity, and we venture to submit the outline of a plan for the purpose, which we think feasible, and which we hope will meet with your sanction and furtherance.

As a nation claiming to lead the world in the science and art of Dentistry, we should have one great representative body of the profession to speak forth with authority its aims, duties, and attainments.

Let us, therefore, organize a National Dental Association of the United States, composed of delegates elected by the various State organizations, an equal number from each State, to meet annually, and always at Washington, D. C. Thus, we would give to Delaware the same voice as to New York. In order to always secure a full meeting, we would suggest six as the number of delegates from each State; a like number of alternates having been elected. Let the National Association elect a Board of Regents, one from each State, who shall control its meetings, setting the time, etc.

As important adjuncts to this Association, we believe it would be found expedient to establish an extensive library, to contain dental works and publications, standard and periodical, foreign and native; and the founding of a national museum, to illustrate the past, present, and future of dentistry.

The wonderful benefit to the profession and humanity in general to be derived from these is certainly manifest to every intelligent dentist.

We are sure that room can be obtained in the Smithsonian Institute for the museum, and in the library of the Surgeon General's office for the library. We are equally sure that Congress will grant an appropriation for both purposes, as is done for the medical profession.

Mature deliberation at the meeting for organizing will suggest the details of the scheme, which we have not thought it necessary to go into here.

In conclusion, we would respectfully urge that you elect, at your present meeting, delegates, with alternates, to meet for the purpose of organization, at the time agreed upon by the chairmen of the different State delegations.

Do this in anticipation of like action upon the part of other State societies; then forward to Dr. B. H. Catching, Atlanta, Ga., the name and address of your chairman, and he will act as medium of communication between the different delegations, informing each of the action of the other, thereby bringing about unity of action. Yours respectfully,

Frank Abbott, New York City.
J. E. Cravens, Indianapolis, Ind.
C. T. Stockwell, Springfield, Mass.
F. Searle, Springfield, Mass.
W. C. Wardlaw, Augusta, Ga.
B. H. Catching, Atlanta, Ga.

